# Aromatic Plants: Antioxidant Capacity and Polyphenol Characterisation

**DOI:** 10.3390/foods6040028

**Published:** 2017-04-04

**Authors:** Charalampos Proestos, Theo Varzakas

**Affiliations:** 1 Laboratory of Food Chemistry, Department of Chemistry, National and Kapodistrian University of Athens, Athens 15771, Greece; 2 Τechnological Educational Institute (TΕΙ) Peloponnese, Department of Food Technology, Antikalamos, Kalamata 24100, Greece; theovarzakas@yahoo.gr

**Keywords:** aromatic plants, HPLC, antioxidant capacity, DPPH, Rancimat test

## Abstract

The antioxidant properties and polyphenol content of some selected aromatic plants grown in Greece were studied. Plants were refluxed with 60% methanol after acid hydrolysis. The phenolic substances were quantified by High Performance Liquid Chromatography–Diode Array Detector (HPLC-DAD). The antioxidant capacity of the extracts was determined with the Rancimat test using sunflower oil as substrate. Free radical scavenging activity was measured using the stable free radical 1,1-diphenyl-2-picrylhydrazyl (DPPH). Results were compared with standard butylated hydroxytoluene (BHT) and ascorbic acid. Total phenol concentration of the extracts was estimated with Folin-Ciocalteu reagent using gallic acid as standard. All plant extracts examined showed antioxidant capacity and contained phenolic compounds. Caffeic acid was detected in all the examined plant extracts. Ferulic acid was also detected in all the methanolic extracts, except from *P. lanata*, in rather high concentration. The amount of total phenolics varied slightly in plant materials and ranged from 8.2 mg to 31.6 mg of gallic acid/g dry sample. The highest amount was found in *O. dictamnus*, and the lowest in *N. melissifolia*.

## 1. Introduction

Aromatic plants are considered as perfect sources of natural antioxidants [1], such as phenolic substances, usually referred as polyphenols, which are ubiquitous components of plants and herbs. Polyphenols are antioxidants with redox properties which allow them to act as reducing agents, hydrogen donators, and singlet oxygen quenchers. Some show metal chelation properties [2,3] and in addition some have antimicrobial activity [4]. A great number of aromatic plants have been reported as having anti-inflammatory, antiallergic, antimutagenic, antiviral antithrombotic, and vasodilatory actions [5].

For example, dictamnus is an aromatic plant found in Crete and it was used from ancient times against stomach pains and as an effective painkiller and wound healing agent. Eucalyptus is one of the most known aromatic plants in the world trade market. It grows in Australia and in Mediterranean countries. Oregano is very often used in Greek cuisine as seasoning due to its strong flavour. It contains glycosides such as apigenin, luteonin, diosmetin [6]. Green tea, such as sideritis, as well as black tea, has been examined by many researchers for their content in polyphenols. In plant materials it is difficult to determine individual flavonoid glycosides. Therefore, the glycosides are hydrolysed and the resulting aglycones are identified and quantified [7].

High performance liquid chromatography (HPLC) is a chromatographic technique which is very common for the simultaneous separation and quantification of phenolic substances. In this study, the identification of each compound was based on a combination of retention time and spectral matching.

The objective of this study was the determination of phenolic compounds in selected plant extracts as well as the evaluation of the antioxidant capacity of the examined extracts in different test systems.

## 2. Materials and Methods

### 2.1. Plant Materials and Reagents

The part of the plants examined and the drying method used are presented in Table 1. Dried samples were obtained commercially or collected from different sites in Greece. All samples were analysed within 3 months of collection. Gallic acid, gentisic acid, p-coumaric acid, vanillic acid, ferulic acid, syringic acid, (+)-catechin, quercetin, apigenin, naringenin, myricetin, were purchased from Sigma-Aldrich (Steinheim, Germany). Luteolin was from Roth (Karlsruhe, Germany). Caffeic acid was from Merck (Darmstadt, Germany). (−)-Epicatechin was from Fluka AG (Buchs, Switzerland). Rutin was from Alexis Biochemicals (Lausen, Switzerland). Ascorbic acid, *p*-hydroxybenzoic acid and BHT (butylated hydroxytoluene) were a kind donation from the National Agricultural Research Foundation (N.AG.RE.F, Greece). Quantification was done via a calibration with standards (external standard method). All standards were prepared as stock solutions in methanol. Working standards were made by diluting stock solutions in 62.5% aqueous methanol containing BHT 1 g/L, and 6 mol/L HCl to yield concentrations ranging between 0.5 mg/L–25 mg/L. Stock/working solutions of the standards were stored in darkness at −18 °C. All solvents and reagents from various suppliers were of the highest purity needed for each application. The Folin-Ciocalteu reagent was purchased from Merck (Darmstadt, Germany). 1,1-diphenyl-2-picrylhydrazyl (DPPH) was obtained from Sigma-Aldrich (Steinheim, Germany).

### 2.2. Extraction Procedure 

The extraction method used for dried samples has as follows: 40 mL of 60% aqueous methanol containing BHT (1 g/L) was added to 0.5 g of dried sample. Then 10 mL of 6 mol/L HCl were added. The mixture was stirred carefully. In each sample nitrogen was bubbled for ca. 40–60 s. The extraction mixture was then sonicated for 15 min and refluxed in a water bath at 90 °C for 2 h. The mixture was then filtered and made up to 100 mL with methanol furthermore filtered quickly through a 0.45 μm membrane filter (Millex-HV, Millipore Corporation, Billerica, MA, USA) and injected to HPLC.

### 2.3. Determination of Total Phenolics

Total phenolic content was measured by the Folin-Ciocalteu assay [8]. Quantification was performed with the hydrolysed samples. Results were expressed as mg of gallic acid/g dry sample.

### 2.4. HPLC Analysis

The analytical HPLC system employed consisted of a JASCO high performance liquid chromatograph coupled with a UV-vis multiwavelength detector (MD-910 JASCO, Tokyo, Japan). The analytical data were evaluated using a JASCO data processing system (DP-L910/V, Mary’s Court, Easton, MD 21601 USA). The separation was achieved on a Waters Spherisorb^®^ 5 μm ODS2 4.6 × 250 mm column at ambient temperature. The mobile phase consisted of water with 1% glacial acetic acid (solvent A), water with 6% glacial acetic acid (solvent B), and water-acetonitrile (65:30, *v/v*) with 5% glacial acetic acid (solvent C). The gradient used was: 100% A 0–10 min, 100% B 10–30 min, 90% B/10% C 30–50 min, 80% B/20% C 50 min–60 min, 70% B/30% C 60–70 min, 100% C 70–105 min, 100% A 105–110 min; post time 10 min before next injection [9]. The flow rate was 0.5 mL/min and the injection volume was 20 μL. The monitoring wavelength was 280 nm for the phenolic acids and 320 and 370 nm (flavones, flavonoles). The identification of each compound was based on a combination of retention time and spectral matching. Linear regression analysis using the least squares method was used to evaluate the calibration curve of each analyte as a function of its concentration. To calculate the reproducibility of the analyses, each phenolic compound was analysed at different concentrations. Results of the HPLC analysis are expressed as the mean of three extractions and triplicate assays. Means, the standard deviation (SDs) and the coefficient of variation (% CV) were calculated. The imprecision was less than 4.0%, apart from luteolin (6.9%). Analytical characteristics of the method are presented in Table 2.

### 2.5. 1, 1-Diphenyl-2-Picrylhydrazyl (DPPH) Scavenging Activity

Experiments were carried out according to the method of Blois [10] with a slight modification. Briefly, a 1 mmol/L solution of DPPH radical solution in methanol was prepared and then, 1 mL of this solution was mixed with 3 mL of sample solutions in different concentrations. After 30 min, the absorbance was measured at 517 nm. The % DPPH radical scavenging is calculated in the equation:

%DPPH radical-scavenging = ((Abs_control_ − Abs_sample_)/Abs_control_) × 100.

The DPPH solution without sample solution was used as control. IC_50_ values of extracts were calculated by using the calibration % DPPH radical-scavenging = f (concentration (μg/mL)) for each plant and expressed in μg/mL.

### 2.6. Rancimat Test

Samples of sunflower oil (3.5 g) containing 0.02% extract or 2% ground material were subjected to oxidation at 110 °C (air flow 20 L/h). Induction periods, IP (h), were recorded automatically. The protection factors (PF) were calculated according to the following formula [11]:

(PF = IP_extract_/IP_control_).

## 3. Results and Discussion

The present method is simple, easy to use, and effective enough for the identification and quantification of major phenolic compounds in aromatic plants. This has been reported by other authors, who have used the same method of extraction and analysis of major Flavonoid aglycones [1,7,12]. Generally, reversed phase HPLC with C_18_ columns is the most popular technique for the analysis of polyphenols in different foods, despite the fact that the separation of procyanidins is not satisfactory with these phases [13]. Spherisorb C_18_ stationary phase, which was used in this study to separate phenolic acids and flavonoids in aromatic plants, produce quite good results. After extraction and acid hydrolysis the content of phenolic substances were determined. Quantification was done via a calibration with standards (external standard method). The amount of phenolic acids detected in the analysed samples is shown in Table 3. Additionally, the content of flavonoids identified in the same plant extracts is shown in Table 4. Results are expressed in mg/100 g dry sample. (+)-Catechin, quercetin and rutin were the most abundant flavonoids. Quercetin has been reported to be present in a large number of aromatic plants [1]. Rutin (quercetin 3-o-rhamnose glycoside) was present along with 22 other flavones, flavonoles and their glycosides in tea leaves [14]. Rutin in some cases can be hydrolysed to quercetin (aglycone). Caffeic acid was detected in all the examined plant extracts. Ferulic acid was also detected in all the methanolic extracts, except from *P. lanata*, in rather high concentration. Phenolic acids are found in nature as esters and rarely as glycosides or in free form [15]. General comments have been published [16,17,18]. Thus, hydrolysis was needed for the identification and quantitative determination of phenolic acids. The data presented in Table 2 and Table 3 are considered as indicative of phenolic content of these aromatic plants. Papers about most of the examined plant extracts are very scarce in the literature. Among others, time of harvest, storage conditions, are considered responsible for the observed variations in the phenolic content.

The antioxidant capacity (expressed as PF values) and the total phenolic content of all extracts are shown in Table 1. The amount of total phenolics varied slightly in plant materials and ranged from 8.2 mg to 31.6 mg of gallic acid/g dry sample. The highest amount was found in *O. dictamnus*, and the lowest in *N. melissifolia*. Similar amounts in plant phenolics from herbs and medicinal plants collected in Finland have been reported recently [8].

The outcome of the Rancimat test supports the hypothesis that aromatic plants are good sources of natural antioxidants such as phenolic compounds. When working accurately, this method offers an efficient, simple and automated assay. When ground material was added to sunflower oil, protection factors were slightly higher compared to the addition of methanol extracts. Similar PF values for ethanol and acetone extracts of plants of Greek origin have been reported [11]. 

**Figure 1:**
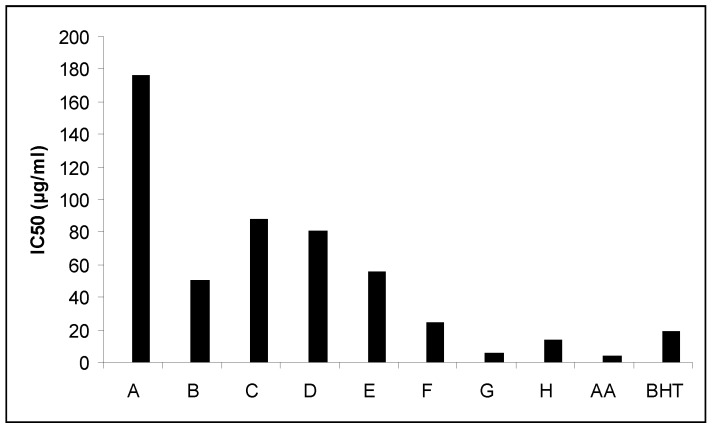
Free radical scavenging activities, where: AA stands for Ascorbic acid; (A) Origanum dictamnus; (B) Eucalyptus globules; (C) Sideritis cretica; (D) Origanum vulgare; (E) Phlomis cretica; (F) Phlomis lanata; (G) Nepeta melissifolia; (H) Mentha pulegium. BHT, butylated hydroxytoluene.

The DPPH method was evidently introduced nearly 50 years ago by Madsen Blois [10], and it is widely used to test the ability of compounds to act as free radical scavengers or hydrogen donors, and to evaluate antioxidant capacity. The parameter EC_50_ (“efficient concentration” value) otherwise called the IC_50_ value, is used for the interpretation of the results from the DPPH method and is defined as the concentration of substrate that causes 50% loss of the DPPH activity (colour). The EC_50_ parameter has the drawback that the higher the antioxidant capacity, the lower is the value of EC_50_. This is a disadvantage particularly when results are presented graphically as a bar chart even if the same data are also available in numerical form. IC_50_ values of *Nepeta melissifolia*, *Mentha pulegium* and *Phlomis lanata* (5.1 ± 0.4, 13.5 ± 0.5, 23.9 ± 0.4 μg/mL) were found to be similar to BHT and ascorbic acid (18.5 ± 0.4, 3.9 ± 0.3 μg/mL). All the IC_50_ values of the examined plant extracts are presented in Figure 1.

## 4. Conclusions

The results in this study indicate that the examined aromatic plants contain certain amounts of polyphenols and their antioxidant capacity was shown. The extracts showed adequate DPPH scavenging activity. Their efficiency toward oxidation of bulk oil under accelerated conditions also produced satisfactory results using the Rancimat apparatus. 

## Figures and Tables

**Table 1:** Total Phenolics in Plant Extracts and their Antioxidant Capacity (Expressed as PF Values).

Family Species	Collection Sites	Part Examined	Drying Method ^a^	Total Phenolics ^b^ (mg Gallic Acid/g ds)	PF ^c^ (Ground Material)	PF (Methanol Extracts)
*Origanum dictamnus*	Crete	Leaves	Air	8.2 ± 0.3	1.3	1.2
*Eucalyptus globules*	Attiki	Leaves	Air	10.5 ± 0.3	1.5	1.4
*Sideritis cretica*	Crete	Leaves	F/v	8.6 ± 0.2	1	1.1
*Origanum vulgare*	Euboea	Leaves	F/v	18.4 ± 0.3	1.8	1.7
*Phlomis cretica*	Crete	Leaves	F/v	16.2 ± 0.1	2.1	1.9
*Phlomis lanata*	Crete	Leaves	F/v	21.4 ± 0.3	2.4	2.1
*Nepeta melissifolia*	Crete	Leaves	F/v	31.6 ± 0.4	3.1	2.9
*Mentha pulegium*	Crete	Leaves	F/v	13.4 ± 0.2	1.9	1.7

**^a^** Air = air drying; F/v = Freeze vacuum, i.e., lyophilization; **^b^** Mean of duplicate assays; ds = dry sample; **^c^** PF = protection factor.

**Table 2:** Analytical characteristics of method for the high performance liquid chromatography (HPLC) analysis.

Phenolic Compounds	Recovery * (%)	Linearity (R2) ^†^	Linearity Rang (mg/L)	Detection Limit ^‡^ (mg/L)	% CV *
gallic acid	89.9 ± 2.6	0.999	0.03–5.97	0.04	3.2
gentistic acid	93.2 ± 2.7	0.998	0.04–5.98	0.04	3.3
Syringic acid	89.9 ± 2.7	0.999	0.03–6.77	0.04	3.2
vannilic acid	93.2 ± 2.7	0.998	0.03–6.75	0.03	4.1
(+)-catechin	98.9 ± 2.9	1.000	0.05–10.12	0.02	3.4
p-coumaric acid	104.3 ± 11.2	0.999	0.05–10.12	0.02	3.6
ferulic acid	101.2 ± 12.5	0.999	0.09–7.21	0.05	3.5
Quercetin	97.5 ± 3.1	0.999	0.09–8.93	0.04	4.6
caffeic acid	87.3 ± 1.6	0.999	0.06–6.45	0.03	3.8
Naringenin	92.5 ± 2.3	0.999	0.07–6.55	0.04	4.4
Luteolin	86.2 ± 2.9	0.998	0.10–6.33	0.18	6.9
Myricetin	90.9 ± 2.4	0.999	0.03–6.75	0.04	3.2
Rutin	92.2 ± 2.7	0.998	0.03–6.75	0.03	3.9
Apigenin	97.8 ± 2.9	1.000	0.05–9.80	0.02	3.3
p-hydroxybenzoic acid	96.3 ± 8.2	0.999	0.05–7.20	0.02	3.5
Eriodictyol	96.8 ± 4.1	0.999	0.09–9.94	0.04	3.8
(−)-epicatechin	95.8 ± 3.1	0.998	0.06–9.85	0.04	3.3

* Results are expressed as mean of three extractions and triplicate assays; CV, coefficient of variation; ^†^ Square of regression coefficient; ^‡^ Three times the noise level.

**Table 3:** Content of phenolic acids in the examined plant extracts (expressed in mg/100 g dry sample ^a^).

Plant	Gallic Acid	Gentisic Acid	Caffeic Acid	*p*-Coumaric Acid	Vanillic Acid	Syringic Acid	Ferulic Acid	*p*-Hydroxybenzoic Acid
*Origanum dictamnus* (A)	4.9 ± 0.03	ND	13.5 ± 0.02	13.9 ± 0.04	18.5 ± 0.02	ND	16.9 ± 0.04	ND
*Eucalyptus globules* (B)	ND	ND	8.1 ± 0.01	6.6 ± 0.02	ND	ND	12.3 ± 0.03	ND
*Sideritis cretica* (C)	1.1 ± 0.02	ND	3.3 ± 0.02	ND	ND	ND	6.8 ± 0.02	2.5 ± 0.01
*Origanum vulgare* (D)	ND	ND	6.4 ± 0.02	ND	ND	ND	10.4 ± 0.03	ND
*Phlomis cretica* (E)	ND	ND	2.2 ± 0.01	ND	ND	ND	5.1 ± 0.02	ND
*Phlomis lanata* (F)	14 ± 0.02	3.2 ± 0.03	20 ± 0.03	4.1 ± 0.02	2 ± 0.02	1.1 ± 0.02	ND	1.5 ± 0.01
*Nepeta melissifolia* (G)	20 ± 0.02	4.3 ± 0.03	26 ± 0.03	5.2 ± 0.02	2.7 ± 0.02	2.6 ± 0.02	22.4 ± 0.03	5.4 ± 0.01
*Mentha pulegium* (H)	ND	ND	13.5 ± 0.02	ND	13.5 ± 0.02	ND	13.5 ± 0.02	ND

^a^ Results are expressed as mean (mg/100 g dry sample ± standard deviation) of three extractions and triplicate assays; ND = not detected.

**Table 4:** Flavonoid content in the examined plant extracts (expressed in mg/100 g dry sample ^a^).

Plant	Quercetin	Apigenin	Luteolin	Naringenin	Myricetin	Rutin	(+)-Catechin Hydrated	(−)-Epicatechin
*Origanum dictamnus*	52 ± 0.09	ND	ND	ND	ND	ND	1.9 ± 0.01	ND
*Eucalyptus globules*	ND	ND	ND	ND	ND	10 ± 0.03	ND	ND
*Sideritis cretica*	ND	ND	ND	ND	ND	ND	6.9 ± 0.02	2.8 ± 0.01
*Origanum vulgare*	7.3 ± 0.02	ND	ND	ND	ND	2.3 ± 0.01	2.5 ± 0.01	ND
*Phlomis cretica*	1.2 ± 0.01	ND	ND	ND	ND	ND	1.5 ± 0.01	2.6 ± 0.01
*Phlomis lanata*	2.2 ± 0.01	ND	ND	ND	ND	4.5 ± 0.01	ND	ND
*Nepeta melissifolia*	11.2 ± 0.02	5.6 ± 0.02	ND	ND	1.8 ± 0.01	2.4 ± 0.01	5.5 ± 0.02	ND
*Mentha pulegium*	ND	6.9 ± 0.02	ND	4.7 ± 0.02	ND	ND	3.5 ± 0.01	ND

^a^ Results are expressed as mean (mg/100 g dry sample ± standard deviation) of three extractions and triplicate assays; ND = not detected.
